# When Glycaemic Control Comes Too Fast: Treatment‐Induced Neuropathy of Diabetes and Charcot Neuro‐Osteoarthropathy as Emerging Iatrogenic Complications of Rapid Metabolic Correction

**DOI:** 10.1002/dmrr.70210

**Published:** 2026-08-20

**Authors:** Dured Dardari

**Affiliations:** ^1^ Diabetology Department Centre Hospitalier Sud Francilien Corbeil‐Essonnes France; ^2^ Laboratoire de Biologie de l'Exercice pour la Performance et la Santé Université d’Evry IRBA Université Paris‐Saclay Evry France; ^3^ Kremlin Bicêtre Medical School Université Paris‐Saclay Kremlin Bicetre France

**Keywords:** Charcot neuro‐osteoarthropathy, early worsening, insulin neuritis, metabolic tempo, microvascular complications, rapid glycaemic correction, treatment‐induced neuropathy of diabetes

## Abstract

**Purpose of Review:**

Diabetes therapeutics can lower glycated haemoglobin (HbA1c) by several percentage points within weeks. While long‐term microvascular risk is reduced by improved glycaemia, abrupt metabolic transitions may destabilise tissues adapted to chronic hyperglycemia. We review treatment‐induced neuropathy of diabetes (TIND) and active Charcot neuro‐osteoarthropathy (CNO) as two neurovascular complications reported after rapid glycaemic correction, and we propose a unifying ‘metabolic tempo’ framework for prevention and early detection.

**Recent Findings:**

TIND is an acute, painful small‐fibre and autonomic neuropathy occurring within 2–8 weeks after large HbA1c reductions, frequently coinciding with early worsening of retinopathy and nephropathy. Human in vivo nerve imaging demonstrates epineurial arteriovenous shunting and proliferative, leaky microvessels, supporting a microvascular dysregulation/ischaemia‐reperfusion model. For CNO, causal evidence linking rapid glycaemic improvement to disease onset remains limited and mainly observational; however, multiple case reports (including pregnancy, post‐transplantation, and major weight loss contexts) and retrospective cohorts suggest that major glycaemic ‘deceleration’ may cluster in the months preceding active CNO in susceptible patients with long‐standing neuropathy.

**Summary:**

Rapid glycaemic correction should not be avoided when urgently needed, but ‘tempo‐aware’ strategies may be warranted in microvascularly fragile patients (very high baseline HbA1c, established neuropathy/retinopathy, kidney disease, or major weight loss). We outline pragmatic risk stratification, surveillance, and interdisciplinary pathways (neurology‐podiatry‐ophthalmology‐nephrology) to reduce delayed diagnosis and prevent deformity and disability.

**Abbreviations**
CGM: continuous glucose monitoring
CN: Charcot neuroarthropathy
CNO: Charcot neuro‐osteoarthropathy
DKD: diabetic kidney disease
DR: diabetic retinopathy
HbA1c: glycated haemoglobin
IWGDF: International Working Group on the Diabetic Foot
OPG: osteoprotegerin
RANKL: receptor activator of nuclear factor‐κB ligand
TCC: total contact cast
TIND: treatment‐induced neuropathy of diabetes

## 1 Introduction

Diabetes care has entered an era of high‐velocity glycaemic correction. Potent incretin‐based combinations, automated insulin delivery, and metabolic surgery can normalise severe hyperglycemia within a short window. For most people this trajectory is beneficial and reduces long‐term microvascular risk. Yet a subset experience acute or subacute ‘early worsening’ phenomena during the first months after abrupt metabolic improvement. Treatment‐induced neuropathy of diabetes (TIND) is an established iatrogenic painful small‐fibre and autonomic neuropathy precipitated by large HbA1c reductions [1]. Early worsening is best described for diabetic retinopathy and has been summarised in recent reviews [2, 3]. Rare but clinically consequential early worsening has also been reported for kidney function in people with chronic severe hyperglycemia [4, 5]. In parallel, an emerging literature proposes that rapid glycaemic regulation may temporally cluster before the onset of active Charcot neuro‐osteoarthropathy (CNO), although causality remains uncertain [6, 7, 8, 9]. Because both TIND and CNO can be disabling and are often missed early, clinicians need an actionable framework to balance urgent glycaemic optimization with short‐term neurovascular safety.

## 2 Methods: Narrative Literature Search

This is a narrative review. Searches were performed in January 2026 using PubMed/MEDLINE and Google Scholar, supplemented by reference‐chaining of key studies and guideline documents. Search terms included combinations of ‘treatment‐induced neuropathy’, ‘insulin neuritis’, ‘acute painful neuropathy’, ‘rapid glycaemic control’, ‘HbA1c reduction’, ‘early worsening’, ‘Charcot’, ‘Charcot neuroarthropathy’, ‘Charcot neuro‐osteoarthropathy’, ‘bariatric surgery’, and ‘pancreas‐kidney transplant’. We prioritised mechanistic studies, clinical cohorts, systematic/narrative reviews, and guideline statements; case reports were used to illustrate rare but instructive presentations and hypothesis‐generating associations.

## 3 Treatment‐Induced Neuropathy of Diabetes (TIND)

### 3.1 Definition and Clinical Phenotype

TIND (historically ‘insulin neuritis’) is an acute iatrogenic neuropathy characterised by the rapid onset of severe neuropathic pain and/or autonomic dysfunction within weeks of a substantial improvement in glycaemic control [1, 10, 11, 12]. The term ‘insulin neuritis' dates back to early case descriptions shortly after the introduction of insulin [13]. In the largest cohort, diagnostic criteria included symptom onset within 8 weeks of a decline in HbA1c of at least 2% points over 3 months [1]. Pain is typically burning and length‐dependent with marked allodynia, often accompanied by weight loss, sleep disruption, and psychological distress [1, 10, 11]. Autonomic features can include orthostatic hypotension, gastrointestinal dysmotility, sweating abnormalities, and genitourinary symptoms [1, 12]. Small‐fibre dysfunction predominates; early nerve conduction studies may be normal [10, 11].

### 3.2 Epidemiology and Risk Factors

Population incidence is unknown, but clinic‐based data suggest TIND is not rare when actively sought. In a tertiary neuropathy cohort, approximately 11% of referred patients met TIND criteria [1]. Risk shows a strong dose‐response relationship with the magnitude of HbA1c reduction: a 2%–3% drop over 3 months corresponded to roughly 20% absolute risk, whereas reductions > 4% over 3 months exceeded 80% absolute risk [1]. Reviews and case series emphasise that the rate of change is central: HbA1c decreases of about 1% per month or more appear to increase risk, particularly in individuals with long‐standing severe hyperglycemia [10, 11, 14, 15, 16]. High baseline HbA1c and long duration of poor control are consistent risk enrichers; TIND is reported more commonly in type 1 diabetes but can occur with any modality that rapidly improves glycaemia (insulin, oral agents, dietary restriction, or metabolic surgery) [1, 10, 11]. Long‐term follow‐up suggests that pain and autonomic symptoms often improve over months, but recovery may be incomplete and may coexist with progression of other microvascular complications [17].

### 3.3 Microvascular and Metabolic Mechanisms

Mechanisms remain incompletely defined. Proposed pathways include endoneurial ischaemia from microvascular dysregulation, ischaemia‐reperfusion injury during abrupt normalisation of hyperglycemia, neuro‐immune activation, and an ‘energy crisis' triggered by sudden shifts in substrate availability in chronically glucose‐adapted tissues [10, 11, 12]. Human in vivo nerve imaging provides direct evidence that vascular biology is central. Using sural nerve epineurial vessel photography and fluorescein angiography, Tesfaye and colleagues demonstrated pronounced microvascular abnormalities in acute painful neuropathy after rapid glycaemic control, including arteriovenous shunting and proliferative epineurial vessels that were leaky to fluorescein (with similarities to retinal neovascularisation) [18]. Earlier work using the same technique in chronic diabetic neuropathy showed impaired perfusion and arteriovenous shunting, supporting a continuum in which diabetic neuropathy is partly a neurovascular disease [19]. These findings provide biologic plausibility for a ‘microvascular steal’ phenotype, in which shunting and capillary leak may reduce effective endoneurial oxygen delivery during metabolic transitions [18, 19].

### 3.4 Systemic Early Worsening: Retina and Kidney

A hallmark of TIND is its association with diffuse microvascular involvement beyond peripheral nerve. In the Brain cohort, retinopathy and microalbuminuria were common and often worsened in the year following TIND onset [1]. Early worsening of diabetic retinopathy after rapid improvement in glycaemia has been described across several therapeutic contexts and summarised in contemporary reviews [2, 3]. Kidney involvement is less well characterised, but early worsening of diabetic nephropathy after rapid improvement in chronic severe hyperglycemia has been reported [4]. More recently, a ‘metabolic descent’ hypothesis has been proposed to explain acute declines in estimated glomerular filtration rate clustering in selected high‐risk patients after rapid correction from severe hyperglycemia [5]. Because modern practice increasingly combines therapies that affect intraglomerular hemodynamics (e.g., renin‐angiotensin blockade, SGLT2 inhibitors, and diuretics) during glycaemic intensification, differentiating expected haemodynamic dips from potentially reversible early worsening related to metabolic tempo is a growing clinical challenge [5].

### 3.5 Diagnosis and Management

Diagnosis is clinical and requires careful temporal correlation between symptom onset and the trajectory of glycaemic correction [10, 11]. Alternative causes of painful neuropathy (diabetic radiculoplexus neuropathy, nutritional deficiencies, toxin exposure, vasculitis, and medication‐induced neuropathies) should be excluded; consensus definitions and phenotyping frameworks can help standardise assessment [20]. Small‐fibre testing (quantitative sensory testing, skin biopsy) can support the diagnosis when uncertainty remains [10, 11]. Management is supportive: neuropathic pain agents (e.g., duloxetine, gabapentinoids, and tricyclics), assessment and treatment of autonomic dysfunction, and mitigation of secondary harms (falls, syncope, and malnutrition) [10, 11, 12, 21]. Whether deliberately loosening glycaemic targets improves outcomes remains uncertain and is not evidence‐based [10, 11]. The most defensible preventive intervention is to anticipate risk and avoid abrupt HbA1c reductions when clinically feasible, while acknowledging that urgent metabolic stabilisation may be necessary in catabolic states [10, 11].

## 4 Active Charcot Neuro‐Osteoarthropathy (CNO)

### 4.1 Clinical Definition and Diagnostic Principles

Active CNO is a destructive inflammatory disorder of bone and joints occurring in a neuropathic limb, most often the foot and ankle in diabetes [9, 22]. The typical presentation is a unilateral warm, swollen, erythematous foot with intact skin and minimal pain, features often misdiagnosed as cellulitis, gout, deep venous thrombosis, or sprain [9, 23, 24]. IWGDF guidance emphasises that active CNO should be suspected in any person with diabetes, neuropathy, and increased foot temperature, oedema, and/or redness compared with the contralateral side [9]. Plain radiographs are first‐line; MRI is recommended when X‐rays are normal to confirm bone marrow oedema and microfracture and to exclude mimics [9, 25]. The key clinical distinctions between TIND and active CNO are summarised in Table 1.

**TABLE 1:** Differentiating TIND and active Charcot neuro‐osteoarthropathy (CNO) in clinical practice.

Feature	TIND	Active CNO
Typical timing	Within 2–8 weeks after large HbA1c reduction [1, 10, 11]	Weeks–months; may follow trauma or metabolic transition [6, 7, 8, 9, 23, 24, 26]
Dominant complaint	Severe burning pain/allodynia; +/− autonomic symptoms [1, 10, 11, 12]	Warm swollen foot; pain often absent or mild [9, 23, 24]
Exam	Small‐fibre signs; large‐fibre deficits may be minimal early [10, 11]	Temperature difference, oedema, and erythema; intact skin common [9, 23, 24, 26]
Key tests	Clinical correlation; small‐fibre testing if needed [10, 11]	X‐ray; MRI if X‐ray normal; thermometry for monitoring [9, 26]
Immediate action	Treat pain/autonomic dysfunction; review glycaemic tempo [10, 11, 12]	Immediate immobilisation/offloading while confirming diagnosis [9, 23, 24, 26]
Major harm if missed	Autonomic morbidity, severe pain, and possible multi‐organ early worsening [1, 2, 3, 4, 5]	Deformity, ulceration, infection, and amputation risk [9, 22, 23, 24, 26]

### 4.2 Pathophysiology: Neuropathy, Inflammation, and Bone Remodelling

Charcot neuro‐osteoarthropathy is now widely conceptualised as an inflammatory syndrome occurring on a substrate of neuropathy, trauma, and dysregulated bone metabolism [22, 23, 24, 27, 28]. Two classic, complementary hypotheses remain useful: the neurotraumatic model (repetitive unperceived trauma in an insensate limb) and the neurovascular model (autonomic dysregulation and hyperaemia promoting bone resorption) [22, 23, 24]. Modern frameworks integrate these with an exaggerated inflammatory response to trauma and dysregulated osteoclastogenesis [22, 27, 28]. Pro‐inflammatory cytokines (including TNF‐α, IL‐1β, and IL‐6) and the RANK‐RANKL‐osteoprotegerin axis are implicated in increased bone turnover and osteolysis [22, 28]. These mechanisms provide plausible interfaces with systemic metabolic transitions that influence endothelial function, inflammation, neurovascular tone, and activity patterns.

### 4.3 Clinical Evidence Linking Rapid Glycaemic Correction to CNO

Evidence connecting rapid glycaemic improvement to the onset of active CNO is heterogeneous and largely observational [6, 7, 8, 26]. Several clinical contexts recurrently appear: (i) intensified diabetes therapy with large HbA1c reductions preceding CNO diagnosis in retrospective cohorts [6, 7]; (ii) pregnancy, where rapid glycaemic optimization and hormonal or biomechanical changes may converge [29]; (iii) major weight loss (dietary or bariatric), where improved glycaemia and increased mobility may coexist with persistent neuropathy [30]; and (iv) pancreas‐kidney transplantation, where abrupt normalisation of glycaemia and glucocorticoid exposure may accelerate bone loss and precipitate CNO [31, 32, 33]. The EPICHAR study and other cohorts observed significant HbA1c declines in the months preceding CNO diagnosis, supporting a temporal association rather than proof of causation [6, 7]. A 2025 narrative review concluded that current evidence does not establish causality and highlighted the scarcity of controlled data and the absence of Charcot reporting in large glycaemic‐lowering trials [8]. Selected clinical evidence linking rapid metabolic correction to neurovascular early‐worsening phenomena is summarised in Table 2.

**TABLE 2:** Selected clinical evidence linking rapid metabolic correction to neurovascular ‘early worsening’ phenomena.

Condition	Study type/setting	Key exposure	Main observation
TIND	Tertiary cohort (Brain 2015) [1]	HbA1c drop ≥ 2% over 3 months	Dose‐response: ≥ 4% drop → very high TIND risk; frequent retinopathy/microalbuminuria worsening [1]
TIND	Mechanistic nerve imaging (Diabetologia 1993; 1996) [18, 19]	Acute painful neuropathy after rapid glycaemic control	Epineurial arteriovenous shunting and proliferative leaky microvessels suggesting endoneurial hypoxia [18, 19]
CNO	Retrospective cohort (PLoS ONE 2020) [6]	HbA1c decline in 6 months pre‐diagnosis	Significant HbA1c reduction preceding acute CNO in a multicenter case series [6]
CNO	EPICHAR study (BMJ Open DRC 2022) [7]	Hyperglycemia correction trend	Suggestive association between HbA1c decline and active CNO onset [7]
CNO	Pregnancy case series (EDM case rep 2020) [29]	Rapid glycaemic optimization in pregnancy	Charcot onset during pregnancy in women with type 1 diabetes with established neuropathy [29]
CNO	Case series after major weight loss (JAPMA 2014) [30]	Improved glycaemia + increased mobility	Charcot onset despite diabetes improvement; persistent neuropathy remains a key risk [30]
CNO	Pancreas‐kidney transplant series [31, 32]	Abrupt normoglycemia + steroids	High early post‐transplant Charcot incidence in selected cohorts [31, 32]
Kidney	Case letter (diabetes care 2021) [4] and hypothesis piece (diabetes metab 2026) [5]	Rapid HbA1c drop from chronic severe hyperglycemia	Acute eGFR decline clustering early after metabolic correction in selected high‐risk patients [4, 5]

### 4.4 Treatment Principles (Active Phase)

The cornerstone of management is immediate immobilisation and offloading to reduce mechanical stress and inflammation while diagnostic confirmation is pursued [9, 26]. IWGDF guidance recommends a knee‐high non‐removable offloading device (often a total contact cast) as first‐line therapy; a knee‐high walker rendered non‐removable is an alternative [9]. Serial skin temperature monitoring (e.g., infrared dermal thermometry) helps track disease activity and guide transition to protected weight bearing [9, 26]. Pharmacologic anti‐resorptive approaches have been explored (including bisphosphonates and RANKL inhibition), but evidence remains inconsistent and these therapies are not recommended for routine use in current guidelines [9, 34, 35]. When offloading fails or deformity/instability threatens skin integrity, reconstructive surgery may be required; recent evidence‐based surgical algorithms synthesise staging and fixation principles [36]. Once inflammation resolves, long‐term prevention centres on protective footwear, activity guidance, and risk‐factor modification [9, 23, 26].

## 5 Integrative Discussion: A Unifying ‘Metabolic Tempo’ Framework

### 5.1 Why the Slope May Matter

Most diabetes risk models—and many quality metrics—prioritise the achieved HbA1c, implicitly treating the path from A to B as clinically neutral. TIND challenges this assumption: in the Brain cohort, the risk of acute painful and autonomic neuropathy rose steeply with larger HbA1c reductions over short intervals, suggesting that the ‘velocity’ of correction can be biologically meaningful [1, 10, 11]. This observation aligns with the broader ‘early worsening’ literature in which abrupt improvement of chronic hyperglycemia can precipitate transient microvascular destabilisation, classically in the retina and increasingly suspected in the kidney [2, 3, 4, 5, 37].

One unifying explanation is that chronic hyperglycemia creates a new microvascular steady state. Structural changes (basement membrane thickening, pericyte loss, and capillary rarefaction), functional adaptations (altered endothelial nitric oxide signalling, impaired vasoreactivity), and neurovascular remodelling (autonomic dysfunction and impaired neurovascular coupling) develop over years. When glucose is lowered rapidly, the system may not recalibrate synchronously: perfusion pressure, plasma osmolality, and local vasodilator/vasoconstrictor tone can shift over days‐to‐weeks, while structural microangiopathy persists for months‐to‐years. The result may be a period of relative hypoperfusion or maldistributed flow (‘functional ischaemia’) in tissues with limited autoregulatory reserve [11, 18, 19].

Additional processes may contribute. Rapid insulin intensification can increase circulating insulin‐like growth factor 1 (IGF‐1), and IGF‐1–linked vascular permeability has been implicated in florid early worsening retinopathy; case reports describe improvement when glycaemic control (and IGF‐1) was deliberately relaxed, highlighting the plausibility of an endocrine permeability signal during abrupt correction [3, 38]. Meanwhile, therapy intensification often increases glycaemic variability and hypoglycemia exposure; hypoglycemia can acutely raise inflammatory mediators such as interleukin‐6, providing a potential systemic inflammatory ‘hit’ that may interact with local microvascular vulnerability [39].

Importantly, the slope of glycaemic correction is (at least partly) modifiable. The central clinical challenge is therefore not whether to improve glycaemia, but how to do so safely in microvascularly fragile patients—without sacrificing the long‐term benefits of durable metabolic control [2, 3, 37].

### 5.2 Candidate Shared Mechanisms Across Nerve, Foot, Eye, and Kidney

A cross‐organ metabolic‐tempo model proposes that rapid correction acts as a physiologic stress test imposed on a microvascular network already remodelled by diabetes. The same patient can experience discordant trajectories across organs: improved symptoms and long‐term risk reduction, yet transient destabilisation in the nerve (TIND), retina (early worsening), kidney (functional eGFR decline), and possibly the neuropathic foot (active Charcot) [1, 2, 3, 4, 5, 6, 7]. Rather than a single mechanism, the phenotype likely reflects convergence of several pathways that can be variably dominant across tissues and individuals.

Microvascular maldistribution and shunting are supported by direct human observations in diabetic neuropathy. Using epineurial vessel photography and fluorescein angiography, Tesfaye and colleagues demonstrated epineurial arterio‐venous shunting, delayed fluorescein appearance, and reduced nerve fluorescence intensity—findings consistent with impaired nutritive flow and potential ‘steal’ phenomena in the vasa nervorum [19]. Related observations described arterio‐venous shunting and proliferating new vessels following insulin initiation (‘insulin neuritis’), suggesting that abrupt metabolic shifts can coincide with dynamic neurovascular remodelling [18].

In the foot, active Charcot is characterised by marked local warmth and hyperaemia, yet perfusion may be heterogeneous and strongly influenced by autonomic dysfunction and inflammation. Maladaptive hyperaemia and/or shunting could reconcile high macroscopic flow with microregional hypoxia, oedema, and cytokine activation, creating a permissive environment for osteoclast‐driven bone resorption and joint collapse [27, 28]. Rapid glycaemic normalisation may contribute indirectly by altering vascular tone, inflammatory signalling, or activity patterns in an insensate limb, as suggested in observational cohorts exploring HbA1c trajectories around Charcot activation [6, 7].

Neurogenic inflammation and innate immune activation may provide a second bridge. TIND involves acute small‐fibre injury with severe pain and autonomic symptoms; inflammatory mediators, oxidative stress, and increased microvascular permeability have been proposed contributors [1, 10, 11]. Active Charcot, in turn, has been linked to elevated proinflammatory cytokines and to RANKL‐mediated osteoclast activation, which amplifies bone turnover once inflammation is established [27, 28]. A systemic inflammatory surge during rapid metabolic transitions—potentially accentuated by hypoglycemia—could therefore lower the threshold for local activation in a susceptible foot or exacerbate neuropathic symptoms [27, 39].

Third, ‘stacking’ of haemodynamic and metabolic perturbations may magnify risk. Rapid glucose lowering is frequently accompanied by weight change, blood pressure reduction, diuretic adjustments, and/or initiation of reno‐protective agents that can cause transient eGFR dips. In vulnerable individuals, the cumulative haemodynamic shift may become clinically relevant and could mirror tempo‐related phenomena observed in nerve and retina [4, 5].

Finally, biomechanical and behavioural mediators should not be underestimated. Resolution of glucotoxic symptoms can increase ambulation, and major weight loss or changes in analgesic use can abruptly change loading patterns in an insensate limb. These factors may unmask subclinical Charcot activity or accelerate structural collapse once inflammation begins, offering a non‐exclusive explanation for temporally clustered cases after metabolic improvement [30].

### 5.3 Alternative Explanations and Sources of Bias

The evidence linking rapid glycaemic correction to active Charcot remains less mature than for TIND, and causal inference is vulnerable to several biases. Much of the literature consists of retrospective series, case reports, and observational analyses in which metabolic intensification coincides with increased surveillance and more frequent diagnostic reassessment [6, 7, 8, 26].

First, detection and ascertainment bias are plausible: therapy intensification increases clinic contact, foot inspection, thermometry, and imaging, which can convert an ‘occult’ Charcot process into a documented diagnosis. Second, confounding by indication is likely: patients selected for aggressive therapy often have very high baseline HbA1c, long‐standing diabetes, severe neuropathy, and advanced microvascular disease—features that increase Charcot risk irrespective of subsequent HbA1c slope [6, 7, 8, 23, 26].

Third, co‐exposures are common and biologically potent. Pregnancy, transplantation, corticosteroids, and rapid weight change each influence bone turnover, immune function, and soft tissue biology and may trigger Charcot independently of glycaemia [29, 31, 32, 33]. Finally, misclassification (e.g., cellulitis, gout, and osteomyelitis) and variable definitions of ‘active’ disease complicate comparisons across studies [9, 26].

These limitations do not negate the tempo hypothesis; they emphasise that rapid glycaemic change should be considered a timing signal and a potentially modifiable co‐factor rather than a proven primary cause. Stronger designs could include prospective cohorts capturing pre‐specified HbA1c slopes, CGM‐derived tempo metrics, and objective Charcot activity measures (temperature asymmetry, MRI bone marrow oedema), with time‐varying adjustment for medications and renal hemodynamics. Within‐patient and contralateral‐foot analyses may further reduce residual confounding [6, 7, 8].

### 5.4 Towards Operational Definitions of ‘Glycaemic Tempo'

Operationalising ‘glycaemic tempo’ requires moving beyond a single HbA1c threshold. HbA1c slope (ΔHbA1c/Δt) remains pragmatic and historically anchored: in the Brain cohort, a decline ≥ 2% over 3 months was associated with a clinically meaningful TIND risk, which escalated sharply with larger drops [1]. However, HbA1c is a retrospective average and does not capture glycaemic variability, hypoglycemia burden, or catabolic reversal, all of which may be relevant to microvascular stress [11].

Future tempo definitions should combine: (i) baseline glycaemic level and long‐term exposure (e.g., HbA1c ≥ 10%–12%); (ii) early rate of change in mean glucose and time‐in‐range from continuous glucose monitoring; (iii) hypoglycemia metrics (time < 70 mg/dL, severe events); and (iv) concurrent physiologic shifts such as rapid weight loss, blood pressure changes, or initiation of therapies with haemodynamic effects (e.g., renin‐angiotensin system blockade, SGLT2 inhibitors) [5, 11].

Because different organs appear to have different vulnerability windows, a time‐anchored approach may help clinicians anticipate risk: TIND often begins within weeks of rapid correction, early worsening retinopathy is commonly reported within 3–6 months, and early nephropathy worsening or functional eGFR decline signals may cluster in the first 6 months [1, 2, 3, 4, 5, 37]. For Charcot, available studies suggest that the active phase can emerge in the weeks‐to‐months following metabolic intensification, but the precise window—and whether a dose–response relationship exists with HbA1c slope—remains uncertain [6, 7, 8, 26].

These considerations argue for a composite ‘tempo risk’ phenotype rather than a binary definition. A clinically useful construct might be tiered risk categories integrating HbA1c slope, neuropathy severity, retinopathy stage, kidney reserve, autonomic symptoms, and expected activity change, which could then trigger tailored monitoring bundles and explicit patient education [1, 2, 3, 9].

### 5.5 Clinical Synthesis: Avoid Therapeutic Inertia, but Respect Microvascular Fragility

Tempo‐aware care is not a rationale for permissive hyperglycemia. Long‐term evidence shows that improving glycaemic control reduces microvascular risk, and early worsening is typically transient and manageable when anticipated and monitored [2, 3, 37]. The clinical goal is therefore not to slow all patients, but to identify the subgroup in whom the immediate physiologic cost of a steep descent may be high—and to mitigate that cost through planning, surveillance, and rapid response to early symptoms.

In high‐risk individuals without a metabolic emergency, stepwise intensification can be paired with explicit counselling about potential short‐term complications (new neuropathic pain, orthostatic symptoms, unilateral hot swollen foot, and visual changes) and with coordinated cross‐organ monitoring. Strategies such as deliberately relaxing glycaemic control to reverse early worsening retinopathy have been reported in exceptional cases, but these approaches are not evidence‐based, may be unsafe, and should not be extrapolated to Charcot or TIND outside specialist oversight [38].

### 5.6 Implications for Contemporary Therapeutics and Quality Metrics

The modern therapeutic landscape enables unprecedented HbA1c declines over weeks using optimised insulin delivery, potent incretin‐based therapies, and continuous glucose monitoring–guided titration. These advances may increase the absolute number of patients exposed to high‐tempo transitions, making rare complications such as TIND and active Charcot more visible in practice [1, 6, 7, 8, 10, 11].

At a system level, treat‐to‐target culture and performance metrics may inadvertently reward speed over safety. Incorporating tempo concepts into care pathways—documenting expected HbA1c trajectory, avoiding multiple simultaneous haemodynamic changes, and deploying structured symptom/temperature checklists—could align quality measures with patient‐centred outcomes while preserving the long‐term benefits of effective glycaemic control [5, 9].

## 6 Practical Implications: Prevention, Early Detection, and Care Pathways

### 6.1 Identify a High‐Risk Phenotype

The goal is not to avoid glycaemic optimization but to individualise the pace and improve surveillance in microvascularly fragile patients. Heightened vigilance is reasonable when one or more of the following are present: very high baseline HbA1c (e.g., ≥ 10–12%); long duration of poor control; established distal symmetric neuropathy; advanced retinopathy; chronic kidney disease; autonomic symptoms; rapid weight loss or catabolic states; pregnancy; or transplantation/steroid exposure [1, 2, 3, 4, 5, 6, 29, 30, 31, 32, 33, 40].

### 6.2 Plan the ‘Descent’ When Clinically Feasible

Where there is no immediate metabolic emergency, clinicians can consider staged intensification rather than maximal multi‐agent escalation in the same short window. As an expert extrapolation (not a guideline), it may be reasonable in very high‐risk individuals to avoid HbA1c drops greater than about 2% over 3 months when feasible, with close follow‐up and iterative titration [1, 10, 11]. In contrast, urgent correction should proceed when clinically necessary (e.g., marked catabolism, ketosis), but should be paired with explicit anticipatory guidance and early surveillance for TIND and CNO [9, 10, 11].

### 6.3 Monitor Early and Across Organs (a ‘Tempo Bundle’)

During the first 8–12 weeks after major glycaemic change, proactive cross‐organ monitoring can reduce delayed diagnosis:

- Nerve/autonomic: ask about new burning pain, allodynia, orthostatic symptoms, gastrointestinal dysmotility, or sudden weight loss; assess hydration and nutrition [10, 11, 12].
- Eye: arrange timely retinal evaluation when large HbA1c reductions are anticipated, especially with pre‐existing retinopathy [2, 3].
- Kidney: monitor creatinine/eGFR and volume status, particularly when initiating therapies that can cause acute haemodynamic eGFR dips [4, 5].
- Foot: perform bilateral temperature and oedema assessment; educate patients to report unilateral warmth, swelling, or redness immediately [9, 23, 26].

### 6.4 Act Fast on Suspected Charcot

Any unilateral hot, swollen foot in a neuropathic patient should prompt immediate immobilisation/offloading and urgent imaging; delays increase deformity and ulceration risk [9, 23, 24, 26]. Notably, TIND‐associated pain can misdirect attention away from the foot, while Charcot often presents with little pain. A low threshold for foot evaluation (including thermometry and MRI when appropriate) is therefore warranted during periods of large glycaemic change [9, 26].

## 7 Research Priorities

Key gaps include: population‐based estimates of TIND and CNO incidence after modern therapies; validated definitions of glycaemic tempo incorporating CGM metrics and HbA1c slope; mechanistic studies of microvascular autoregulation during metabolic transitions; and prospective trials testing whether graded glycaemic correction reduces early worsening without sacrificing long‐term benefit. Pragmatic trials could compare stepped versus rapid correction in individuals with very high HbA1c and established microvascular disease, with predefined neuropathy, retinal, renal, and foot outcomes [1, 9, 26].

## 8 Conclusions

Rapid glycaemic correction is often necessary and beneficial, but it can carry short‐term neurovascular risks in susceptible patients. TIND is an established iatrogenic complication with a clear dose‐response relationship to HbA1c reduction and frequent co‐occurrence of retinopathy and nephropathy progression [1, 2, 3, 4, 5]. For active CNO, evidence linking rapid metabolic improvement to onset is suggestive but not conclusive; nonetheless, the clinical stakes of missed early diagnosis justify heightened awareness during periods of large glycaemic change [6, 7, 8, 9, 23, 24, 26]. Recent case reports continue to highlight that clinically necessary rapid correction can carry substantial neurovascular morbidity, underscoring the need for anticipatory guidance and surveillance [41]. A metabolic‐tempo approach—anticipating risk, staging therapy when feasible, and monitoring nerve, foot, eye, and kidney in parallel—may help clinicians deliver durable metabolic benefit while minimising preventable disability.

## Author Contributions

**Dured Dardari:** conceptualization, literature review, writing – original draft, writing – review and editing.

## Funding

The author has nothing to report.

## Ethics Statement

The author has nothing to report.

## Conflicts of Interest

The author declares no conflicts of interest.

## Data Availability

The data that support the findings of this study are available from the corresponding author upon reasonable request.
